# Functional outcomes of one-stage arthroplasty for native septic knee arthritis: a matched case–control study versus primary osteoarthritis

**DOI:** 10.3389/fcimb.2026.1858682

**Published:** 2026-07-09

**Authors:** Zhenbao Lu, Xiaodan Lin, Tihui Wang, Jianfu Zhu, Jiliang Chen, Xiaolu Wang, Jinqing Wu, Qingshan Xu, Wugui Chen, Chengshou Lin, Xu Wang, Qijin Wang

**Affiliations:** 1 Department of Orthopedics, Affiliated Mindong Hospital, Fujian Medical University, Fuan, China; 2 Department of Neurology, Affiliated Mindong Hospital, Fujian Medical University, Fuan, China

**Keywords:** functional recovery, infection control, one-stage arthroplasty, osteoarthritis, septic arthritis, knee

## Abstract

**Objective:**

To investigate the short-term outcomes of one-stage total knee arthroplasty (TKA) for native septic arthritis of the knee concomitant with osteoarthritis (OA), and to compare these outcomes with those of matched patients undergoing primary TKA for isolated OA during the same period.

**Methods:**

This retrospective study included 11 patients treated between January 2020 and January 2024 for native septic knee arthritis with concomitant OA who underwent one-stage TKA (one-stage group). A contemporaneous control cohort of 33 patients with isolated OA undergoing primary TKA (OA-only group) was selected using individualized 1:3 matching. Perioperative variables, Knee Society Score (KSS), and visual analog scale (VAS) pain scores were compared between groups.

**Results:**

Over a 2-year postoperative follow-up, the infection control rate in the one-stage group was 90.9% (10/11). One patient experienced recurrent postoperative infection, which was controlled after debridement and targeted antimicrobial therapy with implant retention. Postoperatively, KSS clinical and function scores improved significantly and VAS pain scores decreased significantly compared with preoperative values (all *P* < 0.001). Compared with the OA-only group, there were no significant differences in postoperative KSS clinical score, KSS function score, or VAS score (all *P*>0.05). However, operative time, intraoperative blood loss, and postoperative length of stay were significantly greater in the one-stage group (all *P* < 0.001). The overall complication rate did not differ significantly between groups (*P* = 0.812).

**Conclusion:**

In carefully selected patients with native septic knee arthritis concomitant with OA, one-stage TKA may provide substantial functional improvement with a high rate of infection control. Short-term clinical outcomes appear comparable to those of primary TKA for isolated OA, although the perioperative burden is higher.

## Introduction

As the largest synovial joint in the human body, the knee is among the most commonly affected sites of septic arthritis (Mathews et al., 2010; Ross, 2017). Septic arthritis of the knee is most often caused by the invasion of pathogens such as *Staphylococcus aureus* and streptococci into the joint space. If infection control is delayed, rapid and irreversible destruction of articular cartilage and involvement of the subchondral bone may occur, subsequently progressing to secondary osteoarthritis (osteoarthritis, OA). In severe cases, joint deformity and loss of function can ensue (Sharff et al., 2013; Earwood et al., 2021). In recent years, with population aging and the increasing use of intra-articular injections and other invasive procedures, the incidence of septic arthritis of the knee appears to be rising, making its management a major challenge in orthopedic practice (Vidal et al., 2025). For patients with advanced disease concomitant with OA, achieving both complete eradication of infection and effective restoration of joint function remains a key and difficult aspect of clinical decision-making.

At present, the conventional approach for septic arthritis of the knee concomitant with end-stage osteoarthritis generally adopts a staged surgical strategy. Specifically, the first stage involves thorough debridement, joint resection/arthrotomy with temporary joint disuse, and antimicrobial therapy; once the infection is controlled, inflammatory markers have normalized, and local soft-tissue conditions are deemed adequate, a second-stage arthroplasty is performed (Kwon et al., 2025). Although this staged strategy may reduce the risk of PJI at the time of two-stage implantation, it is associated with a prolonged overall treatment course: patients often require multiple operations and episodes of anesthesia, longer hospital stays, and increased healthcare costs (Omoke and Obasi, 2017; Luo et al., 2023). Meanwhile, prolonged immobilization can further lead to joint stiffness, muscle atrophy, and functional limitation, and some patients may ultimately be unable to undergo second-stage reconstruction because of recurrent infection or progression of underlying comorbidities. Moreover, sustained disability not only markedly impairs quality of life but may also increase the risk of complications such as deep venous thrombosis and pulmonary infection (Abram et al., 2020). Therefore, identifying a strategy that can achieve effective infection control while enabling timely functional reconstruction of the joint is of substantial clinical value.

Given the limitations of staged procedures—such as a prolonged treatment course and delayed functional recovery—single-stage arthroplasty has attracted increasing attention in recent years. This approach enables radical debridement, eradication of the infectious focus, and prosthesis implantation within a single operation, which in theory may shorten the overall treatment timeline and reduce the patient’s burden (Sarzaeem et al., 2025). However, in patients with septic arthritis of the knee concomitant with osteoarthritis, single-stage arthroplasty remains challenging in terms of infection control, soft-tissue management, and postoperative functional recovery, and robust evidence supporting its safety and effectiveness is still lacking. Sarzaeem et al. (2025) first reported one-stage primary total knee arthroplasty for acute septic arthritis of the native osteoarthritic knee, suggesting that this approach may be feasible in carefully selected patients. However, comparative data on functional recovery after one-stage arthroplasty in this setting remain limited. Therefore, the present study aimed to evaluate the functional outcomes of one-stage arthroplasty for native septic knee arthritis in comparison with matched patients undergoing primary TKA for osteoarthritis.

As the feasibility, safety, and clinical efficacy of single-stage arthroplasty in patients with septic arthritis of the knee concomitant with osteoarthritis remain to be clarified, we retrospectively reviewed the clinical data of eligible patients who underwent single-stage arthroplasty between January 2020 and January 2024. Outcomes were compared with those of patients with primary osteoarthritis who underwent primary total knee arthroplasty during the same period. We focused on short-term clinical outcomes related to infection control, functional recovery, and complications, with the aim of providing clinical evidence to support the use of this procedure in carefully selected patients.

## Materials and methods

### Study population

This study was approved by the Institutional Review Board/Ethics Committee of our hospital (Approval No. K202603300) and is reported in line with the Strengthening the Reporting of Observational Studies in Epidemiology (STROBE). We conducted a retrospective case–control study including patients admitted to our institution between January 2020 and January 2024 with septic arthritis of the knee concomitant with OA. Patients who underwent single-stage primary total knee arthroplasty (TKA) were assigned to the single-stage TKA group.

Inclusion criteria were as follows: (1) diagnosis of septic arthritis of the knee, with a positive preoperative synovial fluid culture and available antimicrobial susceptibility results; (2) symptomatic, end-stage OA with prior clinical indication for TKA, radiographically confirmed as Kellgren–Lawrence (K–L) grade III–IV; (3) treatment with single-stage primary TKA; (4) complete clinical data and a minimum follow-up of 24 months; (5) acceptable systemic status: hemodynamically stable without systemic sepsis or septic shock, ASA grade ≤ III, controlled diabetes (HbA1c <8.0% and fasting glucose <8.0 mmol/L), and no active intravenous drug use; (6) adequate local conditions: localized infection without extensive extra-articular spread, no severe preoperative flexion contracture (>30°), and satisfactory soft-tissue envelope without extensive ulceration, active sinus tracts, severe scarring or necrosis, or anticipated need for flap coverage.

Exclusion criteria were as follows: (1) negative preoperative synovial fluid culture or unavailable antimicrobial susceptibility results; (2) severe immunocompromised status (e.g., long-term immunosuppressive therapy or HIV infection); (3) hematogenous disseminated or polyarticular infection; (4) severe cardiopulmonary insufficiency or ASA grade > III precluding surgery; (5) poor local soft-tissue conditions (extensive ulceration, active sinus tracts, severe scarring or necrosis, or need for flap coverage) or severe flexion contracture (>30°); (6) a history of prior surgery on the ipsilateral knee.

### Selection of the control group

During the same period (January 2020 to January 2024), patients treated at our hospital for primary knee osteoarthritis were enrolled as the control group (OA-only group); all underwent primary TKA. Patients were individually matched 1:3 to controls by age, sex, BMI, K–L grade, and ASA status.

Inclusion criteria were as follows: (1) diagnosis of primary knee OA with no history of knee joint infection; (2) K–L grade III–IV; (3) treatment with primary TKA; (4) complete clinical data; and (5) a minimum follow-up of 24 months.

Exclusion criteria were as follows: (1) prior ipsilateral knee infection or surgery; (2) revision knee arthroplasty; and (3) inflammatory arthritis, such as rheumatoid arthritis.

### Preoperative pathogen confirmation and antimicrobial susceptibility testing

All patients in the single-stage TKA group underwent a standardized preoperative microbiological work-up. Identification of the causative organism and availability of antimicrobial susceptibility results were considered key prerequisites for performing single-stage TKA. After admission, knee arthrocentesis was performed as early as possible under sterile conditions. Approximately 10–15 mL of synovial fluid was aspirated and inoculated into aerobic and anaerobic blood culture bottles, with an additional aliquot placed in a sterile tube for laboratory testing. Upon receipt, the microbiology laboratory performed Gram staining, bacterial culture, pathogen identification, and antimicrobial susceptibility testing. Susceptibility testing was conducted using the VITEK-2 automated system, and minimum inhibitory concentrations (MICs) were determined. All included patients had definitive preoperative pathogen identification and susceptibility results; patients with negative synovial fluid cultures or without susceptibility data were excluded. Based on susceptibility results, susceptible antibiotics were administered intravenously preoperatively as targeted antimicrobial therapy to enhance the specificity and effectiveness of perioperative infection control.

### Surgical protocol

#### Preoperative preparation

For the single-stage TKA group, an individualized perioperative management plan was developed according to preoperative pathogen identification and antimicrobial susceptibility results. Targeted intravenous antibiotics were initiated preoperatively based on susceptibility testing to improve the precision of perioperative antimicrobial therapy and strengthen infection control. Specific agents were selected according to the organism and susceptibility profile: vancomycin or linezolid was commonly used for methicillin-resistant *Staphylococcus aureus* (MRSA); cefazolin or clindamycin for methicillin-susceptible *S. aureus* (MSSA); ceftriaxone or piperacillin/tazobactam for Gram-negative infections; and combination regimens were used for mixed infections according to susceptibility results. In patients with diabetes, preoperative glycemic optimization was performed, targeting fasting blood glucose <8.0 mmol/L and glycated hemoglobin (HbA1c) <8.0%.

In the OA-only group, preoperative evaluation and perioperative management followed the same principles as the single-stage group, except that infection-related microbiological testing and targeted antimicrobial therapy were not performed. All patients received prophylactic intravenous cefuroxime sodium (1.5 g) 30 minutes before surgery; clindamycin was used in patients with penicillin or cephalosporin allergy.

#### Surgical technique

All procedures were performed by the same senior arthroplasty surgeon. Either general anesthesia or neuraxial anesthesia was used.

In the single-stage TKA group, patients were placed supine. A pneumatic tourniquet was applied to the affected limb and set at 100 mmHg above systolic blood pressure, typically 250–300 mmHg. A standard anterior midline incision (~15 cm) and a medial parapatellar approach were used to expose the knee. Extensive debridement was performed, including removal of inflamed synovium, necrotic cartilage, devitalized bone, and intra-articular purulent material. Specimens of synovium, subchondral bone, and synovial fluid were collected intraoperatively for bacterial culture (and susceptibility testing when necessary), and findings were interpreted in conjunction with preoperative microbiology. The joint was then irrigated sequentially with 3% hydrogen peroxide, 0.5% povidone–iodine, and copious normal saline using pulse lavage. This sequence was repeated for three cycles, with a total lavage volume of approximately 9–12 L. After debridement, the wound was temporarily closed; the surgical field was re-prepped and re-draped, and surgical instruments, gloves, and relevant sterile items were replaced before proceeding with prosthesis implantation. A posterior-stabilized fixed-bearing knee prosthesis was implanted. Antibiotic-loaded bone cement was prepared using an antibiotic selected according to the preoperative pathogen and susceptibility results (e.g., vancomycin 2.0 g for MRSA). Depending on intraoperative findings, one or two closed-suction drains were placed, and the wound was closed in layers with a compressive dressing.

In the OA-only group, primary TKA was performed following the standard procedure, without infection-focused debridement. Antibiotic-loaded cement was not used. Synovial tissue was routinely sent for bacterial culture to exclude occult infection.

#### Postoperative management

Single-stage TKA group: Postoperatively, intravenous susceptible antibiotics were continued according to preoperative microbiology and susceptibility results for at least 2 weeks. When infection-related markers such as C-reactive protein (CRP) and erythrocyte sedimentation rate (ESR) had markedly decreased and largely normalized, and remained stable on two consecutive reassessments, therapy was switched to an orally administered susceptible antibiotic with high bioavailability for an additional 6 weeks. The total duration of antimicrobial therapy was typically 8 weeks, adjusted according to infection control status. Closed-suction drainage was routinely used and removed 24–48 hours postoperatively based on drainage volume and wound condition; the removal criterion was <50 mL over 24 hours. Rehabilitation started on postoperative day 1 with quadriceps isometric exercises and ankle pump exercises. After drain removal, continuous passive motion (CPM) and active knee flexion–extension exercises were initiated. Partial weight-bearing ambulation with a walker began at 1 week postoperatively, progressing to full weight-bearing over 2–4 weeks. Body temperature, wound healing, and infection-related laboratory indices were closely monitored; complete blood count, CRP, and ESR were rechecked weekly until normalization and stability on two consecutive tests.

OA-only group: Prophylactic antibiotics (cefuroxime sodium) were administered routinely for 24 hours postoperatively. Drain management and the rehabilitation protocol were the same as in the single-stage group.

Susceptible intravenous antibiotics were commenced immediately upon receipt of antimicrobial susceptibility results, with pathogen-specific regimens as follows: vancomycin or linezolid for MRSA; cefazolin or clindamycin for MSSA; penicillin G or ceftriaxone for Streptococcus; vancomycin or cefazolin for coagulase-negative staphylococci based on oxacillin susceptibility; ceftriaxone, piperacillin-tazobactam, or ceftazidime for Gram-negative bacilli per susceptibility; and combination therapy for mixed infections. Transition to oral therapy (e.g., linezolid, levofloxacin, or clindamycin) was permitted once CRP and ESR had decreased by >50% toward normal limits, the patient was afebrile for ≥48 hours, wounds were dry, and a highly bioavailable oral agent was available. The total antimicrobial duration was 8 weeks (typically 2 weeks intravenous followed by 6 weeks oral), adjusted only if inflammatory markers failed to normalize.

### Outcome measures

#### Primary outcomes

Infection eradication was defined using a composite endpoint adapted from the MSIS PJI definition (Parvizi et al., 2011) and the 2021 EBJIS definition endorsed by MSIS (McNally et al., 2021), modified for native septic arthritis: (i) clinical resolution—absence of wound drainage, sinus tracts, local inflammatory signs, fever >38.0°C, or infection-related pain; (ii) laboratory resolution—serial CRP and ESR trending to normalization (CRP <10 mg/L and ESR <20 mm/h, or return to pre-infection baseline) with stability on two consecutive weekly tests; (iii) microbiological resolution—no need for antibiotic suppression beyond the planned 8-week course, and negative cultures from any clinically indicated aspiration; and (iv) radiographic stability—no progressive osteolysis, new peri-implant radiolucent lines >2 mm, or bone destruction. Infection recurrence was defined as the reappearance of any of these criteria after initial normalization, necessitating re-intervention or prolonged antibiotic therapy.

Knee function: Knee function was assessed using the Knee Society Score (KSS), including the KSS clinical score and KSS function score. The KSS clinical score primarily evaluates pain, range of motion, and stability, whereas the KSS function score mainly evaluates walking ability and stair climbing (Insall et al., 1989). Assessments were performed preoperatively and at 3, 12, and 24 months postoperatively, as well as at the final follow-up.

Pain: Pain intensity was evaluated using the Visual Analogue Scale (VAS) (Langley and Sheppeard, 1985), ranging from 0 to 10 points, where 0 indicates no pain and 10 indicates severe pain.

#### Secondary outcomes

Perioperative variables: Operative time, intraoperative blood loss, and postoperative length of hospital stay were recorded. Intraoperative blood loss was calculated based on the volume collected in the suction canister and the gravimetric method (gauze weighing).

Radiographic evaluation: Standard anteroposterior and lateral knee radiographs were obtained immediately after surgery and during follow-up to evaluate prosthesis position, lower-limb alignment and the presence of prosthetic loosening, subsidence, or migration. Prosthetic loosening was defined as a radiolucent line >2 mm in width or progressive widening of a radiolucent line on serial radiographs.

Complications: Postoperative complications were recorded, including infection recurrence, wound-healing problems (e.g., delayed healing and superficial infection), deep vein thrombosis, pulmonary embolism, periprosthetic fracture, and neurovascular injury.

### Statistical analysis

All analyses were performed using SPSS version 26.0. Continuous variables were tested for normality using the Shapiro–Wilk test. Normally distributed data are presented as mean ± standard deviation (mean ± SD). Pre- versus postoperative comparisons within groups were performed using paired-samples *t* tests, and between-group comparisons were performed using independent-samples *t* tests. Categorical variables are presented as frequency (percentage) and were compared using the chi-square test or Fisher’s exact test, as appropriate. Time effects of KSS and VAS scores between the two groups were analyzed using repeated-measures analysis of variance. A two-sided *P* value <0.05 was considered statistically significant.

## Results

### Patient inclusion flow, baseline characteristics, and matching

From January 2020 to January 2024, 16 patients with septic knee arthritis were treated at our institution. According to the inclusion and exclusion criteria, 5 patients were excluded (2 had negative preoperative synovial fluid cultures, 1 had uncontrolled diabetes (HbA1c >8.0%), 1 had severe immunocompromised status, and 1 had prior ipsilateral knee surgery), and 11 patients were ultimately included in the one-stage arthroplasty group. Using a 1:3 individualized matching approach, 33 patients undergoing primary TKA for OA-only during the same period were matched as the control group.

There were no significant differences in preoperative baseline characteristics between the two groups (all *P*>0.05), indicating comparability (**Table 1**). All 11 patients in the one-stage arthroplasty group successfully underwent surgery. No intraoperative deaths or major neurovascular injuries occurred during the perioperative period. Compared with the OA-only group, the one-stage arthroplasty group had a longer operative time, greater intraoperative blood loss, a longer postoperative hospital stay and higher intraoperative transfusion rate; these differences were statistically significant (*P* < 0.001, *P* < 0.001, *P* < 0.001 and *P* = 0.014, respectively) (**Table 2**).

**Table 1:** Comparison of preoperative demographics between the two groups.

Parameters	One-Stage(n=11)	OA-only(n=33)	P value
Age, yrs (SD)	67.5±5.2	66.8±4.9	0.802^a^
Sex (M/F, n)	5/6	12/21	0.592^b^
BMI, kg/m² (SD)	25.8±3.4	26.0±3.1	0.791^a^
Affected side (left/right, n)	4/7	15/18	0.772^b^
Kellgren-Lawrence grade (III/IV, n)	3/9	10/23	0.517^b^
Preoperative KSS clinical score (SD)	42.5±8.3	43.2±7.8	0.797^c^
Preoperative KSS functional score (SD)	45.2±9.1	46.1±8.6	0.773^c^
Preoperative VAS score	7.2±1.5	6.9±1.4	0.543^a^
Preoperative range of flexion (°, SD)	84.2±14.6	86.3±13.8	0.669^c^
Diabetes mellitus (n)	5	10	0.303^b^

^a^ Independent-samples t-test.

^b^ Chi-squared test.

^c^ Mann-Whitney U test.

**Table 2:** Comparison of perioperative parameters between the two groups (mean±SD).

Parameters	One-Stage (n=11)	OA-only (n=33)	P-value
Operative time (min)	125.0±28.5	73.2±11.6	<0.001^a^
Intraoperative blood loss (mL)	385.5±102.4	188.3±56.7	<0.001^b^
Postoperative hospital stay (d)	16.2±2.1	5.9±1.3	<0.001^a^
Intraoperative transfusion (n)	4	1	0.014^c^

^a^ Independent-samples t-test.

^b^ Mann-Whitney U test

^c^ Chi-squared test

Preoperative synovial fluid cultures were positive in all 11 patients in the one-stage arthroplasty group: *Staphylococcus aureus* in 5 cases (45.5%), including 1 case of MRSA; coagulase-negative staphylococci in 3 cases (27.3%); *Streptococcus* spp. in 2 cases (18.2%); and Gram-negative bacilli in 1 case (9.1%). Intraoperative tissue culture results were fully concordant with the preoperative synovial fluid culture results (100% agreement).

### Prosthesis survival analysis

During the 2-year postoperative follow-up, infection did not recur in 10 patients in the one-stage arthroplasty group. One patient developed recurrent infection at 4 weeks postoperatively, presenting with wound erythema and swelling, drainage, and a renewed elevation in C-reactive protein (CRP). Infection was controlled after debridement, antibiotics, and implant retention (DAIR).

**Figure 1:**
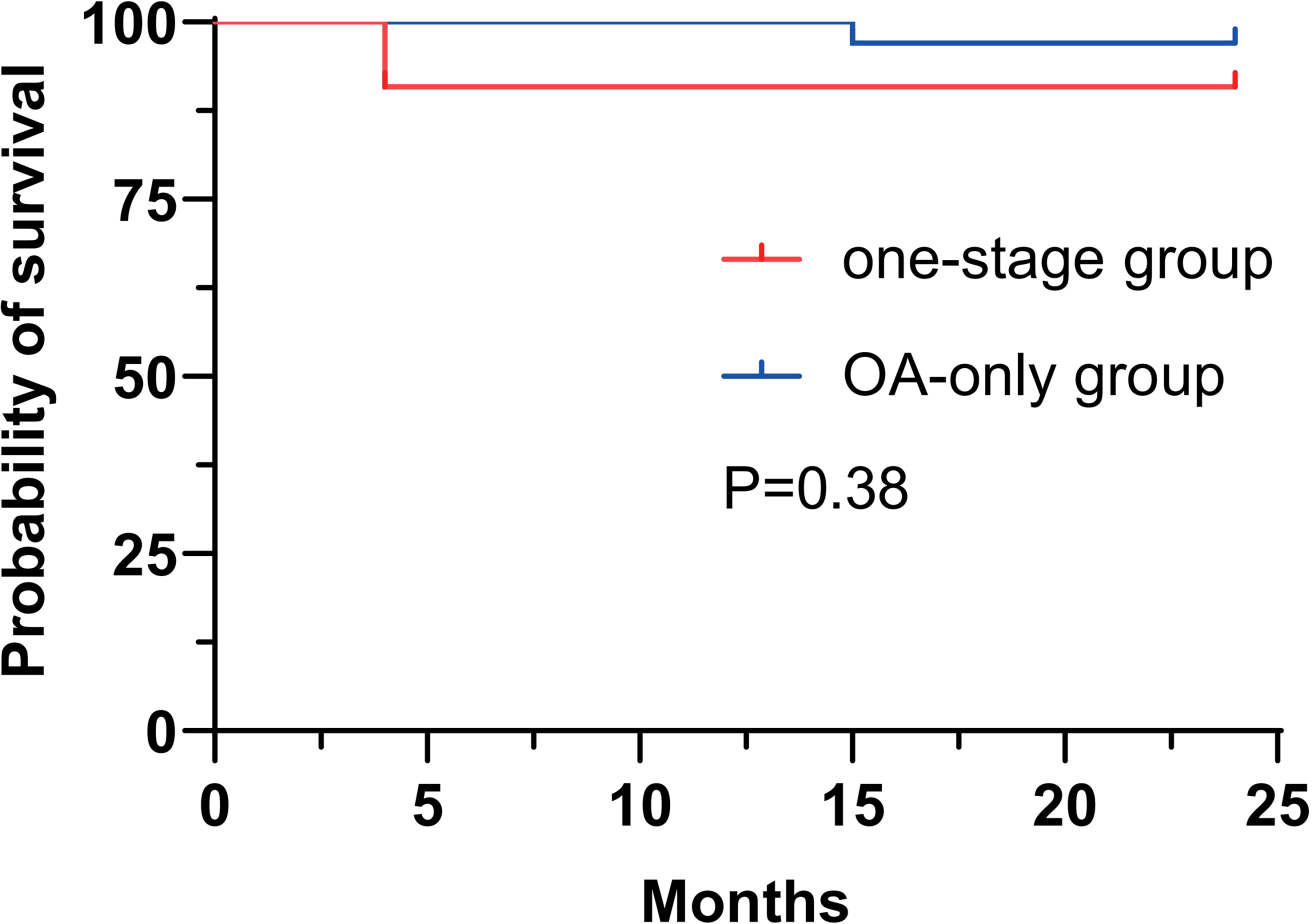
Kaplan–Meier survivorship curves comparing implant survival at 2-year follow-up between the one-stage arthroplasty group for septic knee arthritis and the primary OA group.

In the OA-only group, one patient developed tibial-sided loosening secondary to trauma at 15 months after surgery and ultimately underwent revision. Kaplan–Meier survival analysis showed that prosthesis survival at 24 months in the one-stage arthroplasty group was comparable to that in the OA-only group (**Figure 1**).

### Functional recovery and comparison with the OA-only group

At 24 months postoperatively, the one-stage arthroplasty group demonstrated marked improvements in knee function and pain compared with the preoperative status. The KSS clinical score, KSS function score, VAS score, and range of motion were all significantly improved relative to baseline (all *P* < 0.001) (**Table 3**).

**Table 3:** Functional outcomes before and after surgery: One-stage group (n=11, mean±SD).

Variables	Preoperative	24-month follow-up	P-value
KSS Clinical Score, pts	42.5±8.3	84.5±7.6	<0.001^a^
KSS Functional Score, pts	45.2±9.1	82.3±8.7	<0.001^a^
VAS Score, 0–10	7.2±1.5	2.2±0.9	<0.001^a^
Knee Flexion, degrees	84.2±14.6	105.5±12.3	<0.001^b^
Extension Deficit, degrees	12.8±5.5	2.5±1.8	<0.001^a^

^a^ Independent-samples t-test.

^b^ Mann-Whitney U test

Compared with the OA-only group, overall functional recovery at 24 months was similar in the one-stage arthroplasty group, with no significant between-group differences in postoperative KSS clinical score, KSS function score, KSS improvement, or VAS score (all *P*>0.05) (**Table 4**).

**Table 4:** Comparison of functional indices between the two groups at 24 months postoperatively (mean±SD).

Variables	One-Stage(n=11)	OA-only (n=33)	P-value
KSS Clinical Score, pts	84.5±7.6	85.8±7.1	0.612 ^a^
KSS Functional Score, pts	82.3±8.7	84.1±8.2	0.547 ^a^
VAS Score, 0–10	2.2±0.9	1.9±0.8	0.314 ^a^

^a^ Independent-samples t-test.

### Complications and representative case

Postoperative complications occurred in 2 patients (18.2%) in the one-stage arthroplasty group and in 5 patients (15.2%) in the OA-only group, with no significant difference between groups (*P* = 0.812) (**Table 5**). No serious complications, such as deep vein thrombosis, pulmonary embolism, periprosthetic fracture, or permanent neurovascular injury, were observed.

**Table 5:** Comparison of complications between two groups during 24-month follow-up.

Complications	One-Stage(n=11)	OA-only(n=33)	P-value
Recurrent infection	1	0	-
Poor wound healing	1	4	-
Deep vein thrombosis (DVT)	0	0	-
Pulmonary embolism (PE)	0	0	-
Periprosthetic fracture	0	0	-
Aseptic loosening	0	1	-
Neurovascular injury	0	0	-
Overall complications	2	5	0.812 ^a^

^a^ Chi-squared test.

**Figure 2:**
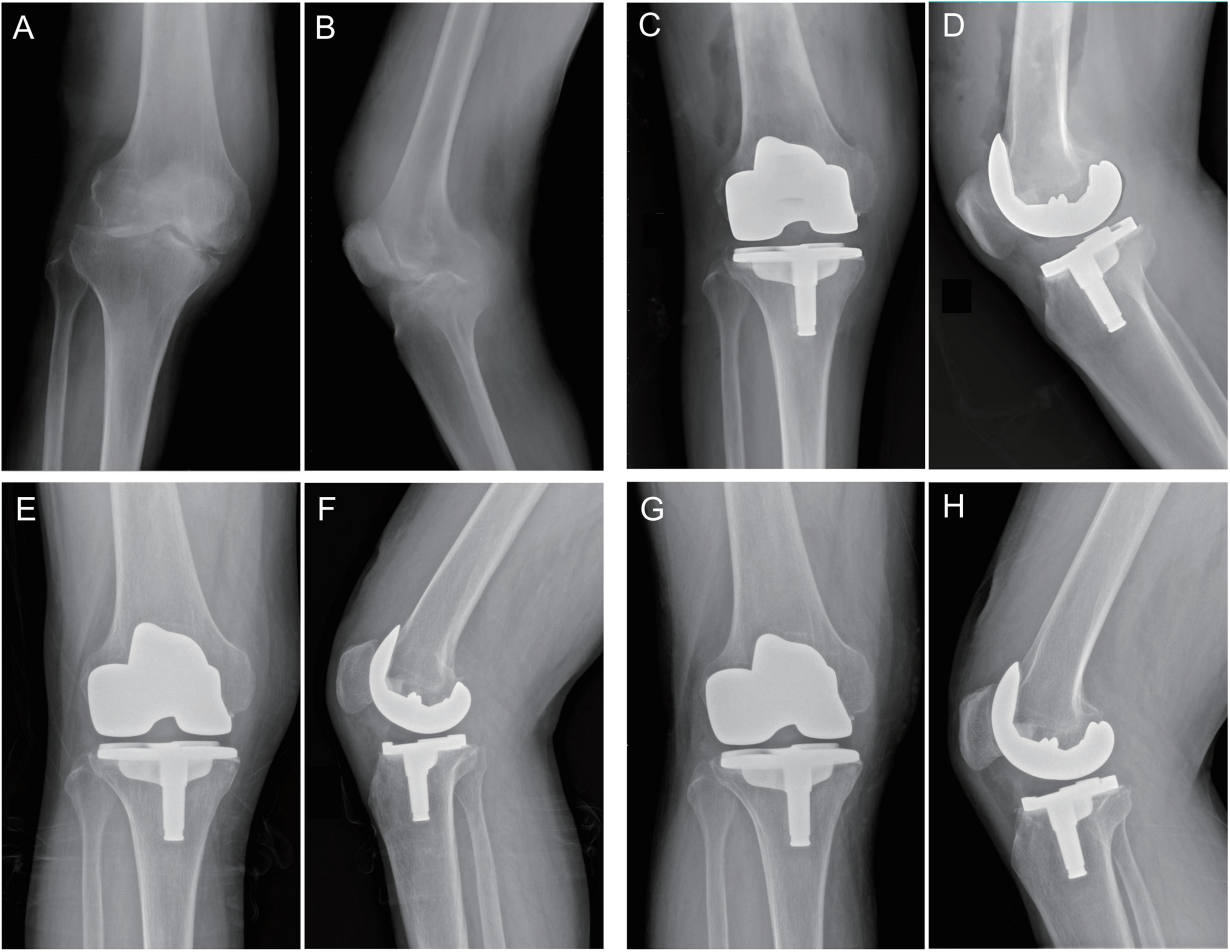
Serial radiographs of the right knee. **(A, B)** Preoperative anteroposterior (AP) and lateral views. **(C, D)** Postoperative radiographs at 3 months. **(E, F)** Postoperative radiographs at 12 months. **(G, H)** Postoperative radiographs at 24 months, demonstrating satisfactory implant positioning.

Representative case: A 62-year-old woman with an 8-year history of left knee OA and a 35-day course of septic infection. Synovial fluid culture yielded coagulase-negative staphylococci. She underwent one-stage TKA. At 24 months postoperatively, her KSS clinical score was 82 points, with satisfactory functional recovery (**Figure 2**).

## Discussion

The present study showed that, in a strictly selected cohort of patients with septic knee arthritis complicated by end-stage osteoarthritis, one-stage arthroplasty achieved a high final infection control rate during follow-up (90.9%). Moreover, at 24 months postoperatively, both the KSS clinical score and KSS function score were comparable to those of contemporaneous patients undergoing primary TKA for osteoarthritis alone. These findings suggest that, provided that indications are carefully defined and perioperative infection assessment and management are standardized, one-stage arthroplasty may represent a feasible treatment option for this specific patient population.

Traditionally, septic knee arthritis complicated by advanced OA has been treated with a staged surgical strategy, typically involving initial debridement and/or spacer implantation followed by second-stage arthroplasty after infection control (Ni et al., 2020). Romanò et al. reported that two-stage arthroplasty for septic arthritis can achieve satisfactory infection control (Romanò et al., 2011). However, Anagnostakos et al., in a two-stage protocol for septic arthritis of the hip, observed an initial infection control rate of 87% (13/15) after the first-stage spacer implantation, which increased to 100% after repeat spacer exchange, but the mortality rate after completion of the first stage was 18% (Anagnostakos et al., 2016). In addition, spacer-related complications (e.g., soft-tissue contracture, loss of muscle strength, and limb-length alteration) may increase the technical difficulty of reimplantation and compromise final functional recovery (Chen et al., 2008). Yong-Uk Kwon et al. similarly showed that although two-stage TKA can effectively control infection and improve function, it is associated with higher rates of periprosthetic joint infection (12% vs 0%) and reoperation (16% vs 1.3%) than conventional primary TKA for OA, along with comparatively poorer functional recovery (Kwon et al., 2025). Recent evidence indicates that two-stage exchange for periprosthetic joint infection is associated with substantial failure accrual beyond the first postoperative year, with survivorship declining from 77% at 1 year to 38% at 5 years in mixed cohorts, underscoring the necessity for extended follow-up to capture late recurrence and aseptic loosening (Shannon et al., 2026). Portier et al. reported the largest cohort of arthroplasty following native septic arthritis to date (49 mixed hip and knee joints, 88% one-stage), observing that new periprosthetic joint infections with organisms distinct from the initial pathogen can occur even in the absence of septic-arthritis relapse (Portier et al., 2022). In the present study, the final infection control rate achieved with one-stage arthroplasty was broadly comparable to outcomes reported for staged strategies. Moreover, by completing joint reconstruction in a single operation, this approach may shorten the overall treatment course and potentially reduce problems related to prolonged functional limitation and muscle atrophy.

Direct evidence on primary one-stage arthroplasty as an initial treatment for septic arthritis of the knee remains limited, possibly because this strategy has traditionally been considered to carry a higher risk of infection recurrence and to have relatively restrictive indications. In recent years, however, with improvements in perioperative infection assessment, thorough debridement, and antimicrobial strategies, an increasing number of reports have described single-stage procedures that achieve both infection control and joint reconstruction. Sarzaeem et al. reported three patients with osteoarthritis complicated by acute septic arthritis who underwent single-stage total knee arthroplasty; at 2-year follow-up, symptoms and function improved and no infection recurrence was observed (Sarzaeem et al., 2025). Hooper et al. performed one-stage joint reconstruction using a metal–polyethylene articulating spacer in patients with native knee infection and concomitant degenerative joint disease, reporting an implant retention rate of 83% and favorable functional outcomes, with overall results not inferior to those achieved with the traditional two-stage strategy using a static spacer (Hooper et al., 2021). Building on these findings, the present study further included patients undergoing primary TKA for osteoarthritis alone as a control group. The results showed that, at 24 months postoperatively, the one-stage arthroplasty group achieved KSS clinical and functional scores comparable to those of the control group, suggesting that—when patients are strictly selected and infection management is standardized—one-stage arthroplasty may yield a level of functional recovery approaching that of conventional primary TKA.

This study emphasizes that obtaining definitive preoperative microbiological evidence and implementing pathogen-directed antimicrobial therapy accordingly are key prerequisites for achieving favorable outcomes with a one-stage arthroplasty strategy. For all included patients, specimens were collected before initiation of antibiotic therapy, and positive culture results with complete antimicrobial susceptibility profiles were obtained. Based on these data, individualized antibiotic regimens were developed: intravenous administration of susceptible agents was started 24 h preoperatively to ensure adequate systemic antimicrobial coverage at the time of surgery; antibiotic-loaded bone cement incorporating susceptible antibiotics was used intraoperatively to increase local drug concentrations and enhance local antibacterial activity; and an 8-week course of postoperative systemic anti-infective therapy was completed according to the protocol. This management pathway is consistent with the fundamental principles of treating periprosthetic joint infection—namely, “pathogen identification, targeted therapy, thorough debridement, and a standardized treatment duration.” Prior studies have likewise indicated that a definitive preoperative microbiological diagnosis facilitates selection of effective antibiotics and reduces the risk of recurrence (Omoke and Obasi, 2017; Kildow et al., 2020; Yi et al., 2025). In the present study, the combination of preoperative targeted antibiotics and intraoperative antibiotic-loaded cement provided dual systemic and local antimicrobial barriers. The 100% concordance between intraoperative and preoperative cultures further supports the accuracy of the microbiological assessment.

Thorough intraoperative debridement is a key component of one-stage arthroplasty for septic arthritis of the knee. In the present study, the one-stage procedure involved extensive excision of inflamed synovium and debridement of necrotic cartilage and bone, combined with 9–12 L of pulsatile lavage, to minimize the residual infectious burden as much as possible. Compared with the OA-only group, the one-stage arthroplasty group had a longer operative time (approximately 2 h vs approximately 70 min), which may be attributable to the broader extent of debridement and the additional irrigation steps. Peersman et al. previously reported that, in conventional TKA, an operative time exceeding 127 min was associated with an increased risk of postoperative infection (Peersman et al., 2006); however, this evidence was derived primarily from patients undergoing primary TKA in a non-infectious setting. For procedures in which infection eradication is the primary objective, a longer operative time may, to some extent, reflect a more comprehensive debridement and treatment workflow, and its clinical implications should be interpreted in conjunction with infection control outcomes and perioperative complications.

In this study, one patient (9.1%) developed recurrent infection at 4 weeks postoperatively; infection was successfully controlled with DAIR, and implant removal was not required. This recurrent case was caused by MRSA. Previous studies have shown that, among patients with periprosthetic joint infection, infections due to methicillin-resistant organisms are associated with relatively lower infection control rates after two-stage revision and have been considered a risk factor for reinfection (Santoso et al., 2020). However, evidence remains limited regarding patient selection and risk stratification for “primary arthroplasty performed for septic arthritis.” The single MRSA recurrence in our cohort (9.1%) suggests that methicillin-resistant organisms should be regarded as a high-risk criterion for one-stage TKA in native septic arthritis. However, given the small cohort, these findings cannot establish organism-specific risk profiles, and this recurrence may reflect patient-specific or host factors in addition to pathogen virulence. Based on this observation and the available literature, the indication for one-stage arthroplasty should be evaluated cautiously in patients with infection caused by resistant organisms such as MRSA, those with extensive bone loss, or those with poor soft-tissue conditions. When appropriate, a staged surgical strategy may be considered to improve the likelihood of infection eradication and reduce the risk of recurrence.

One of the main findings of this study was that postoperative functional recovery in the one-stage arthroplasty group was broadly comparable to that in patients undergoing primary TKA for isolated OA, suggesting a potential functional advantage of one-stage arthroplasty when appropriate indications and standardized perioperative management are applied. In contrast, staged procedures may be complicated during the interim period by prolonged immobilization or an “interval arthroplasty-free” state, leading to joint stiffness and muscle atrophy; consequently, even after successful infection control, functional recovery may remain limited in some patients. Haddad et al. reported higher postoperative KSS scores after one-stage revision than after two-stage revision (88 vs 76), with a 100% infection control rate in their cohort (Haddad et al., 2015). In the present study, the early postoperative rehabilitation protocol was essentially the same as that used in the OA group: CPM and active functional exercises were initiated immediately after drain removal and were not routinely delayed because of the infectious context, with the aim of reducing the risks of stiffness and loss of muscle strength. A systematic review by Nagra et al. further indicated that, with strict case selection, infection control with one-stage revision can be comparable to that achieved with a two-stage strategy (overall reinfection rate, 4.3% vs 13.5%), while potentially yielding superior functional outcomes by avoiding interval-related stiffness and muscle atrophy (Nagra et al., 2016). Taken together, the available evidence and our findings suggest that one-stage arthroplasty may offer a more favorable balance between infection eradication and functional recovery; however, its applicability should still be determined on an individualized basis, taking into account factors such as microbiological characteristics, soft-tissue conditions, and the extent of bone loss.

Several limitations should be acknowledged. First, this retrospective, small-sample study was underpowered for robust stratified/subgroup analyses by pathogen, bone loss, or soft-tissue status and is susceptible to selection bias and residual confounding. Second, as a single-center cohort, its perioperative protocols and surgical expertise are center-specific, which may limit generalizability. Third, follow-up was relatively short, capturing mainly short-term infection control and functional recovery, and was insufficient to assess late recurrence, aseptic loosening, or long-term implant survival. Fourth, the absence of a contemporaneous 2-stage control group limits our ability to directly compare infection control strategies. Future studies should ideally include a 2-stage arm to determine whether one-stage TKA offers non-inferior infection control with superior functional recovery. Finally, the study was powered for functional outcomes, not for rare complications or long-term prosthesis survival; thus, these secondary findings should be interpreted cautiously.

## Conclusions

In this strictly selected, single-center cohort with 24-month follow-up, one-stage arthroplasty for native septic knee arthritis with concomitant osteoarthritis was associated with satisfactory short-term infection control and functional improvement. These preliminary, hypothesis-generating findings suggest that one-stage TKA may be feasible in carefully selected patients with standardized perioperative management. However, no direct comparison with two-stage septic TKA was performed, and conclusions regarding relative efficacy cannot be drawn. Confirmation through larger, multi-center studies directly comparing one-stage and two-stage protocols is required.

## Data Availability

The datasets presented in this article are not readily available. Requests to access the datasets should be directed to qijinwang1940@163.com.
